# Supplementary material for: SQANTI3: curation of long-read transcriptomes for accurate identification of known and novel isoforms
Source: Nat Methods. 2024 Mar 20;21(5):793–7. doi: 10.1038/s41592-024-02229-2 (PMC11093726; doi:10.1038/s41592-024-02229-2)
Supplement: Supplementary file 1 — Supplementary Notes 1–5, Supplementary Methods. [file 41592_2024_2229_MOESM1_ESM.pdf]

nature methods

Brief Communication https://doi.org/10.1038/s41592-024-02229-2

SQANTI3: curation of long-read transcriptomes for accurate identification of known and novel isoforms

In the format provided by the authors and unedited

Contents

1 Supplementary Notes **2**

1.1 Supplementary Note 1. Main differences and upgrades across SQANTI software versions . . . 2

1.2 Supplementary Note 2. SQANTI3 supports Functional Iso-Transcriptomics (FIT) analysis: a hESC differentiation example 4

1.3 Supplementary Note 3. In-depth analysis of the TSS and TTS variability in the WTC11 PacBio transcriptome . . . 11

1.4 Supplementary Note 4. Analysis of SQANTI3 Filter performance under a variety of input-data scenarios . . . 16

1.5 Supplementary Note 5. Application of SQANTI3 pipeline to a dRNA ONT defined transcriptome . . . 20

2 Supplementary Methods **24**

2.1 SQANTI3 QC . . . 24

2.1.1 STAR . . . 24

2.1.2 Kallisto . . . 24

2.2 Running IsoSeq3 from subreads to transcriptome . . . 25

2.2.1 CCS . . . 25

2.2.2 lima and refine . . . 25

2.2.3 IsoSeq3 cluster and collapse . . . 25

2.3 Running SQANTI3 QC for the WTC11 transcriptome . . . 26

2.3.1 Reference genome and annotation . . . 26

2.3.2 Short-read support . . . 26

2.3.3 PolyA motif list . . . 26

2.4 Running SQANTI3 QC for the Low Input (LI) scenario . . . 26

2.5 Running SQANTI3 QC for High Input Sample (HIS) and Reference (HIR) scenarios . . . 27

2.6 Running SQANTI3 QC for the K562 transcriptome . . . 27

2.7 Running SQANTI3 QC for the H1-endoderm transcriptome . . . 27

2.8 Running SQANTI3 QC for reference transcriptomes . . . 28

1 Supplementary Notes

1.1 Supplementary Note 1. Main differences and upgrades across SQANTI software versions

Among its many contributions, the initial SQANTI software featured the definition of structural categories for the classification of isoforms. These were created mainly based on the characteristics of splice junctions (SJ), with two main categories capturing junction-level similarity to the reference transcriptome (FSM, ISM), and two others referring to different aspects of junction-level novelty (NIC, NNC). While SQANTI2 maintained this classification scheme, SQANTI3 introduced several subcategories for FSM and ISM, aiming to additionally capture TSS and TTS-related novelty. This allows for a more thorough understanding of the isoform catalog, as well as for the design of better transcriptome curation strategies, as was demonstrated in the main text of this manuscript.

Similarly, the quality control (QC) process enabled by the first SQANTI release was largely based on short-read coverage of the junctions, with many of the computed quality features being associated with this data source. However, this precluded the validation of novel TSS and TTS, and prevented users from removing unreliable FSM and ISM transcripts, which were often fully supported at the SJ level. To mitigate this, the integration of region-level data (CAGE, Quant-seq, polyA sites) and reliable polyA motifs was implemented in both SQANTI2 and SQANTI3. This was extended via the development of a novel TSS support metric, the TSS ratio, as part of SQANTI3. Importantly, the fact that the TSS ratio is computed using short-read coverage at the 5' end allows users lacking region-level data to perform TSS validation, and may complement CAGE data retrieved from databases in cases where novel TSS are highly sample-specific. As a result of this implementation, SQANTI3 now takes raw short-read data as input, which does not require users to map and quantify to generate the required inputs for QC.

Another feature included in the initial SQANTI that has been significantly refactored is the machine-learning (ML) filter. The first major change constitutes the incorporation of the CAGE, polyA, and TSS ratio features for classifier training, which enables automated filtering of ISM and FSM transcripts. Previously, the hard-coded usage of all FSM as the True Positive (TP) set and the lack of TSS and TTS-related QC attributes precluded the removal of false positive FSM and ISM transcripts. The SQANTI3 ML filter further solves this by allowing users to provide a custom list of TP and TN transcripts, whereas subcategories, i.e. Reference Match (RM) FSM transcripts, are used to build the default TP set when required. In addition to these improvements, users are further empowered to perform flexible and comprehensive transcript curation thanks to the novel Rules Filter and Rescue modules in SQANTI3. Supplementary Table 1 provides a comparative feature chart across SQANTI versions.

Supplementary Table 1. Comparative feature chart across SQANTI versions

		SQANTI	SQANTI2 (unpublished)	SQANTI3
QC	Main SQANTI categories	✓	✓	✓
	Subcateogries	✗	✗	✓
	SQANTI QC descriptors	✓	✓	✓
	Processed short-reads	✓	✓	✓
	Raw short-reads	✗	✗	✓
	CAGE peaks	✗	✓	✓
	polyA sites	✗	✓	✓
	polyA motifs	✗	✓	✓
	Aligners	GMAP	minimap2, deSALT	minimap2, GMAP, deSALT, uLTRA
	TSS ratio	✗	✗	✓
	IsoAnnotLite	✗	✗	✓
	QC report	✓	✓	✓
ML filter	Usage of SQANTI QC descriptors	✓		✓
	Usage of orthogonal data	✗		✓
	User-defined class sizes for training	✗		✓
	Automated filtering of output files (FASTA, GTF...)	✗		✓
	Exclusion/inclusion mono-exon transcripts	✗		✓
	Removes low-quality FSM and ISM	✗		✓
	Filtering report	✗		✓
Rules filter	Attributes used for filtering		SJ coverage, intra-priming, diff. annotated TTS	All attributes
	Thresholds by structural category		✗	✓
	Allows logical rules		✗	✓
	Filtering report		✗	✓
Rescue		✗	✗	✓

1.2 Supplementary Note 2. SQANTI3 supports Functional Iso-Transcriptomics (FIT) analysis: a hESC differentiation example

The SQANTI3 framework offers not only quality control and curation but also the integration of IsoAnnotLite, which allows for isoform-level functional annotation. This feature facilitates downstream analyses on isoform biology, for example, using the tappAS software [1]. To demonstrate this capability, we selected a human Embryonic Stem Cell (H1-hESC) dataset in which H1 cells had been differentiated *in vitro* into human Definitive Endodermal (DE) cells. These two cell cultures were sequenced using both Illumina short-read sequencing and PacBio lrRNA-Seq as part of the LRGASP project. Long-read data for these samples is available at ENCODE accessions ENCSR271KEJ (H1) and ENCSR127HKN (DE). Long reads from all samples and replicates were pooled to reconstruct a single, experiment-specific transcriptome using IsoSeq3 and cDNA Cupcake for collapse (see Methods of main document). To perform QC using SQANTI3, Illumina data was retrieved from ENCODE accessions ENCSR588EJX (H1) and ENCSR266XAJ (DE). The refTSS database was to retrieve human TSS annotations, while TTS support was computed by combining a human polyA motif list (supplied within SQANTI3) and 52,558 peaks from sample-specific Quant-seq data supplied by the LRGASP consortium (ENCODE accession: ENCSR198UNH).

After IsoSeq3 and Cupcake processing, a total of 211,107 transcript models were initially detected, 89% of which belonged to 17,667 known genes. Data from the refTSS database and Quant-seq data, as well as same-sample short reads and a list of known human polyA motifs, were used to remove potential artifacts using the SQANTI3 ML filter (see Main paper Methods). After filtering and rescue (see Main paper Methods), 65,255 transcript models from 14,541 known genes were included in the annotation (Supplementary Figure 1.**a**). Among them, there were 4,043 single-isoform and 10,498 multi-isoform genes (Supplementary Figure 1.**b**). We used stringent filtering for potential fragments and isoforms with novel SJ, resulting in a curated transcriptome that was enriched in FSM and NIC, and had few ISM and NNC transcripts (Supplementary Figure 1.**c**).

A total of 4,247 (29.25%) genes and 21,489 (36,2%) transcripts were found to be significantly Differentially Expressed (DE, FDR < 0.05) when comparing hESC and endoderm. This analysis recapitulated the behavior of known markers of differentiation[2], namely the downregulation of OCT4, SOX2, and NANOG in endoderm and the upregulation of FOXA2, SOX17, and GATA4 (Supplementary Figure 2.**a**).

For the functional annotation of the H1-DE dataset, the SQANTI3 QC script was re-run using the post-rescue transcriptome as input and the `--isoAnnotLite` and `--gff3` arguments. The functionally-annotated GENCODE v39 reference transcriptome available at the tappAS website (https://app.tappas.org/) was supplied to the `--gff3` flag. This annotation included 9 transcript-level and 11 protein-level feature types. A tappAS-compatible

GFF3 file was obtained as a result. The vast majority of isoforms (82.7%) were functionally annotated with at least one functional element, being UTRsite motifs [3] (86.2% of transcripts annotated) and PFAM domains (90% of predicted CDS annotated) the most frequently transferred terms (Supplementary Figure 2.**b**). Short read-based quantification of isoform expression was then performed for each sample and replicate, and both isoform quantification and annotation data were used as input for tappAS analysis of H1-hESC versus endoderm differences.

Using tappAS' Functional Diversity Analysis (FDA [1]), we observed that the majority of multi-isoform genes showed changes in the inclusion of transcript (50%) and protein (70%) features in at least one isoform, indicating that a high amount of predicted functional features were associated with potential changes in isoform functionality (Supplementary Figure 2.**c**). To understand the contribution of Alternative Splicing (AS) to this process, the tappAS´s Differential Isoform Usage (DIU) analysis was applied. We detected 450 genes with significant DIU (FDR < 0.05), revealing a weaker contribution of AS to the divergences between both differentiation stages than that of DE genes. These genes were enriched in ubiquitination sites, RNA recognition domains, and Nuclear Localization Signals (adjusted p-value < 0.05, Supplementary Figure 2.**d**), suggesting a splicing-based regulation of the inclusion of these functional elements.

To identify genes in which alternative splicing was likely to be the main regulatory mechanism, we selected candidates that exhibited significant DIU (FDR < 0.05) but no differentiation-induced changes in gene-level expression between the two cell stages. The ACOX2 gene was found among the DIU genes with the highest isoform usage change (Supplementary Figure Supplementary Figure 2.**e**) while also showing no differential gene expression (Supplementary Figure 2.**f**). This gene encodes the Acyl-CoA Oxidase 2 enzyme, responsible for fatty acid degradation in the peroxisome [4]. At the structural level, the major isoform in the H1 cell line lacked the first 9 exons, which resulted in the H1-specific loss of the N-terminal and central peroxisomal Acyl-CoA oxidase protein domains (Supplementary Figure 2.**g**). Both domains were diferentially excluded in the H1-hESC sample (DFI adjusted p-value < 0.05). Upon differentiation into endoderm, the major isoform switch caused the long isoform to be almost uniquely expressed, leading to a condition-specific inclusion of the three PFAM domains necessary for full functionality.

The ETS1 transcription factor was another interesting example of development-related changes in the transcriptomic landscape that would be masked if the gene-level expression were considered exclusively. ETS1 is a gene known to be involved in cell development [5] and did not exhibit significant variation in the H1 transition H1 from hESC to endoderm. However, significant changes in transcription start site (TSS) usage were detected when analyzing isoform expression differences between the two cell types (DIU p-value < 0.05), which ultimately changed coding sequence (CDS) length (Supplementary Figure 3.**a-c**). The shorter protein (UniProt ID:P14921-1), highly

expressed in endoderm, includes two Lys-Gly isopeptides that interact with SUMO2, while the longer isoform (UniProt ID:P14921-3), highly expressed in H1, showed exclusion of these sites (Supplementary Figure 3.**d**). Our analysis suggests that this isoform switch in the ETS1 gene may involve the gain of the ability to bind a post-transcriptional modifier during differentiation, which can constitute a splicing-mediated regulation of the role of this transcription factor.

By using long-read RNA sequencing (lrRNA-seq) to generate the transcriptome, we were also able to identify functionally relevant genes that undergo DIU involving isoforms not described in the reference annotation. For instance, the Chromodomain Y-like (CDYL) gene, which encodes a chromatin reader protein involved in gene activation and repression via chromatin change [6], was found to have up to five distinct isoforms identified by lrRNA-seq. Two of these isoforms were NIC by the combination of known splice sites and differed from each other only at the 3'UTR (Supplementary Figure 4.). The remaining 3 isoforms were FSM of ENST00000397588.8 (Reference Match and Alternative 3' end) and ENST00000472453.5 (Alternative 3' end). The most highly expressed isoforms in H1 and endoderm were PB.21000.1 (longest NIC) and PB.21000.6 (FSM Reference Match), respectively (Supplementary Figure 4.**a**). The usage of an alternative first exon by the novel isoform PB.21000.1 in H1 resulted in the exclusion of the chromodomain at the N-terminus (DFI adjusted p-value < 0.05), which was re-gained in endoderm after the isoform switch with the domain-including FSM PB.21000.6 (Supplementary Figure 4.**b-c**). This isoform switch may have implications for the activity of CDYL during cell differentiation, possibly involving a splicing-regulated increase in the ability of this protein to change chromatin architecture. These results emphasize the significance of augmenting the reference annotation with transcript models acquired from lrRNA-seq. Moreover, they demonstrate that when combined with functional annotation, lrRNA-seq has the potential to detect novel isoform switches of potential biological relevance.

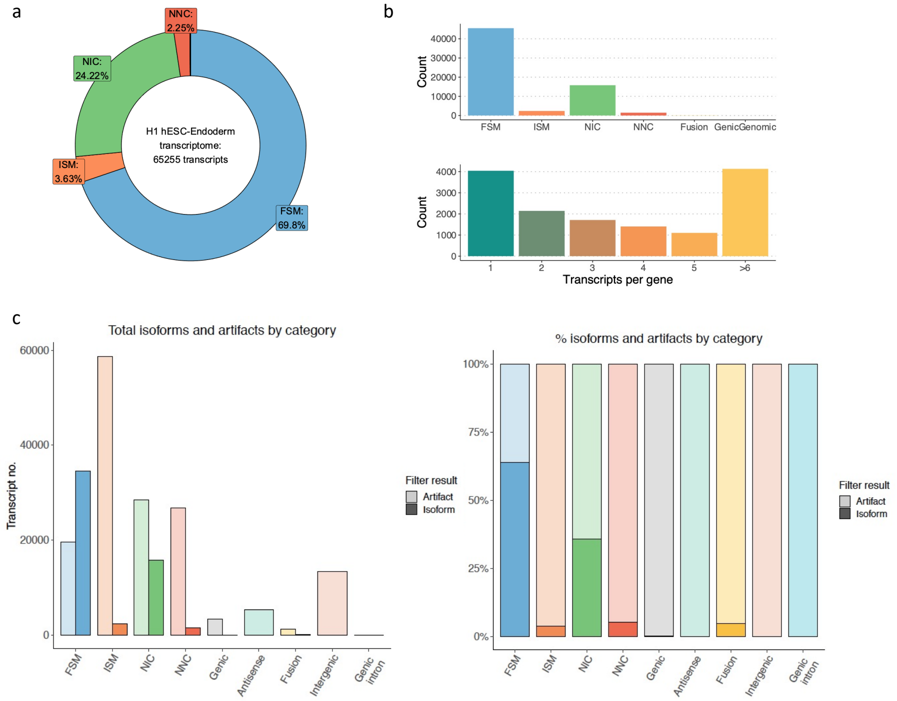

Supplementary Figure 1. Characterization of hESC differentiation transcriptome. a and **b**, Isoform distribution across structural categories of the long-read defined H1 transcriptome. **c**, Distribution of isoforms and artifacts by structural category identified in the hESC H1-endoderm differentiation experiment, after running an ML-based filter. Absolute number of isoforms classified as isoforms or artifacts per structural category and percentage of isoforms and artifacts within each structural category.FSM: Full-Splice-Match, ISM: Incomplete-Splice-Match, NIC: Novel-In-Catalog, NNC: Novel-Not-In-Catalog, ML: Machine Learning.

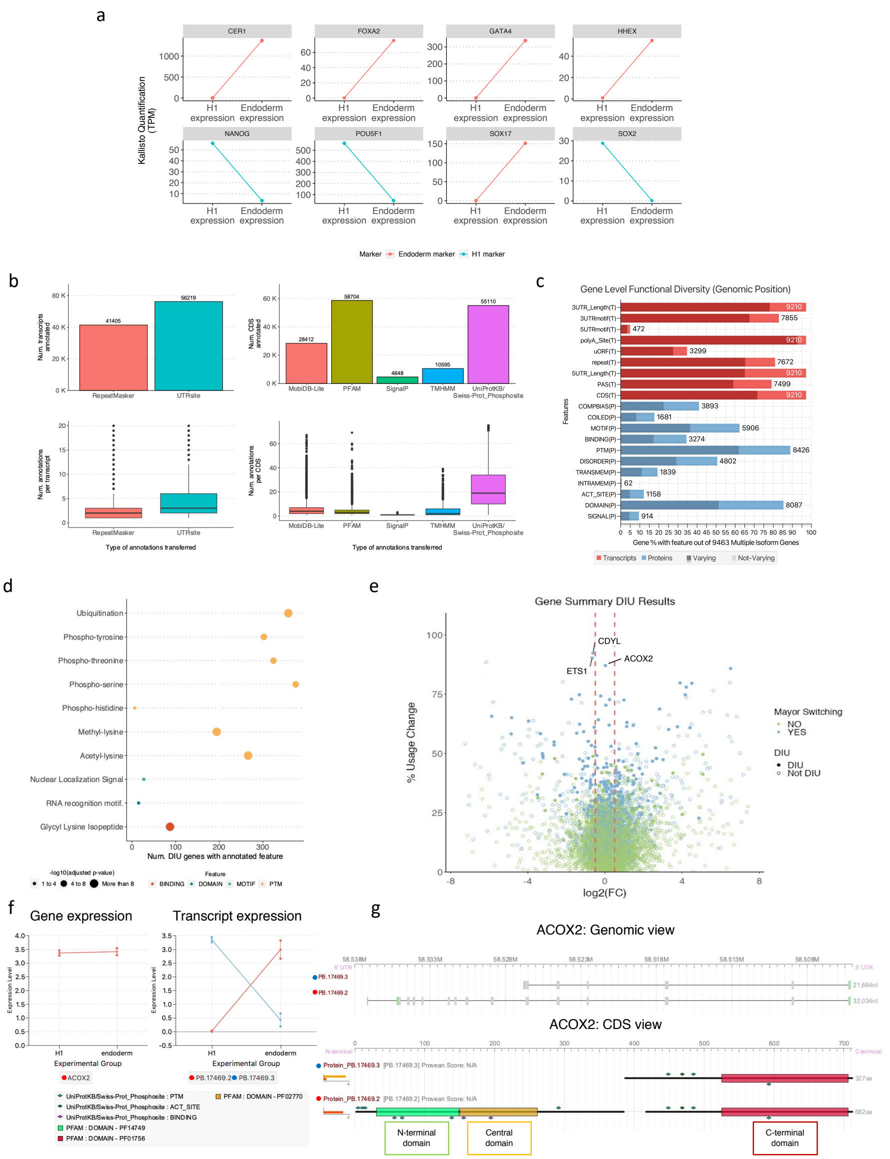

Supplementary Figure 2. Functional IsoTranscriptomics Analysis of the H1 hESC to endoderm differentiation. **a** Gene expression (TPM) of hESC H1-endoderm differentiation markers calculated using Kallisto. **b** Functional annotation success for transcripts and CDS. Boxes indicate median (middle line), 25th (Q1) and 75th (Q3) percentiles (box hinges); whiskers represent min $= Q1 - 1.5 \cdot$ Interquartile Range (IQR) and max $= Q3 + 1.5 \cdot$ IQR; dots constitute outliers. **c** Level of annotation of multi-transcript genes by isoAnnotLite and analyzed with tappAS **d**, Functional Enrichment Analysis performed by tappAS [1] of annotated features in those genes with Differential Isoform Usage compared to Diferentially Expressed genes. **e**, Percentage of Isoform Usage Change of identified genes (y-axis) compared to their expression fold-change in expression (x-axis). **f**, Mean expression (+/- SD) at the gene and transcript levels for ACOX2. **g**, Genomic and CDS views of the identified isoforms for the ACOX2 gene, including functional features annotated with IsoAnnotLite.

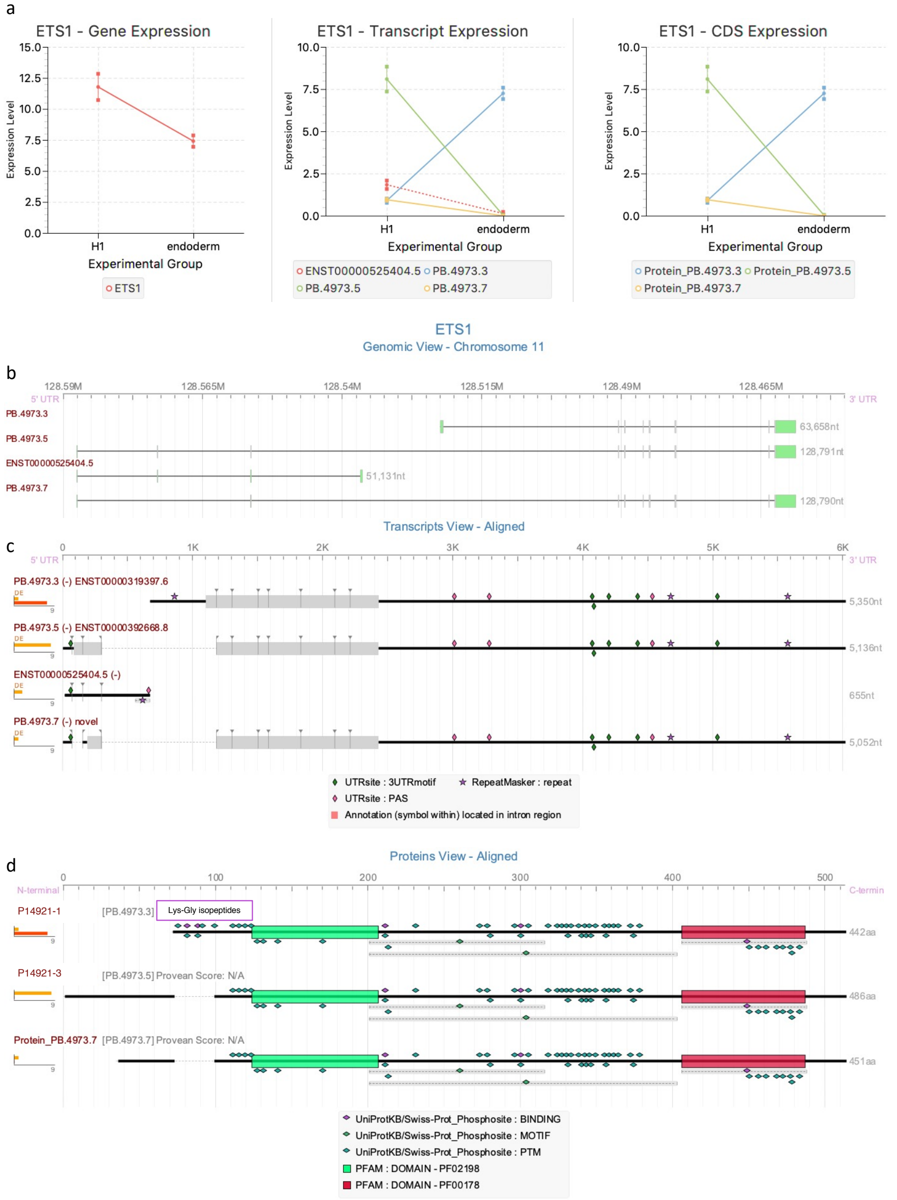

Supplementary Figure 3. tappAS visualization of ETS1 gene through hESC H1-endoderm differentiation. A) Mean expression (+/- SD) values (TPM) at the gene, transcript, and CDS levels. The dotted line in the transcript expression plot represents a non-coding isoform. B) Genomic View. Green segments represent untranslated regions. C) Transcript View. Thick sections represent the location of predicted ORFs. D) Protein View.

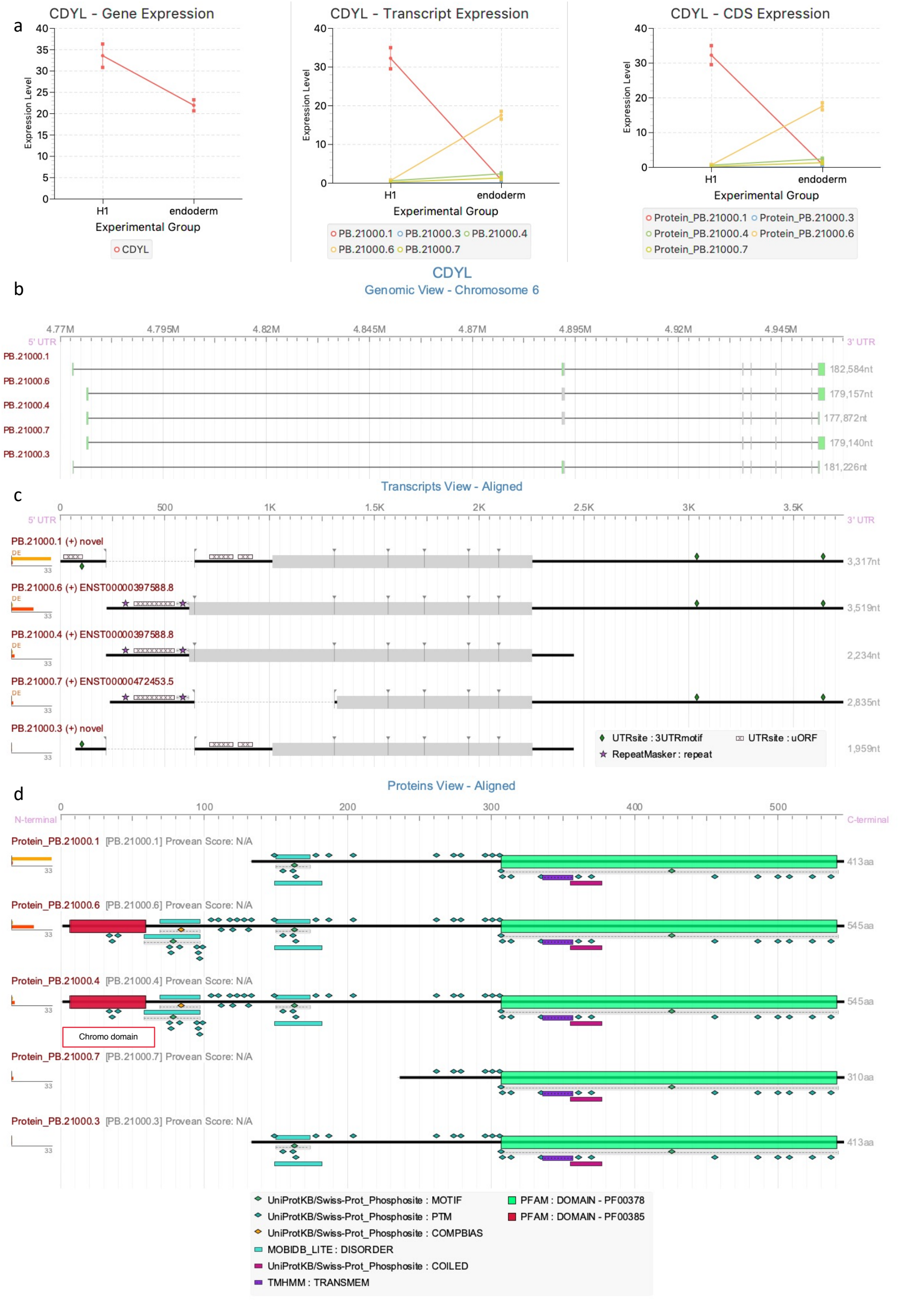

Supplementary Figure 4. tappAS visualization of CDYL gene through hESC H1-endoderm differentiation. A) Mean expression (+/- SD) values (TPM) at the gene, transcript, and CDS levels. B) Genomic View. Green segments represent untranslated regions. C) Transcript View. Thick sections represent the location of predicted ORFs. D) Protein View.

1.3 Supplementary Note 3. In-depth analysis of the TSS and TTS variability in the WTC11 PacBio transcriptome

In-depth characterization of the variability at 5' and 3' ends of long-read transcript models in the WTC11 sample is facilitated by analyzing their distribution across SQANTI3 subcategories. We found that only 22.5% (12,805 out of 56,795) of FSM were Reference Match (RM) transcripts, while 58.8% of them showed variation at 3´ends (belonged to either the Alternative 3´ end or Alternative 5´/3´end subcategories) (Supplementary Figure 5.). Complementary evidence indicated that these end sites were validated in 39.7%, 67.9%, and 87.8% of cases by a same-gene annotated TTS, Quant-seq data, and the presence of a polyA motif, respectively. A similar pattern was observed for TSS within the Alternative 5' end subcategories: 47.2% fell within an alternative annotated start site, 66.2% were supported by CAGE-seq, and 70.9% had a TSS ratio greater than 1.5 (Supplementary Figure 5.). The subcategory-level analysis of FSM, therefore, reveals incompleteness in the reference annotation and suggests that novel combinations of known start/end sites and intron chains are yet to be described.

The analysis of ISM subcategories showed that 3' Fragment (3'F) was by far the most abundant group with a total of 47,594 transcript models (70.2% of ISM), where only 9.4% had a known TSS, 16% had CAGE-seq support and 39.5% displayed an above-threshold TSS ratio (Supplementary Figure 5.). This pattern was recapitulated by the 13,236 mono-exonic ISM transcripts, for which most 3' ends were validated by orthogonal data, whereas 5' ends remained largely unsupported. Moreover, transcripts from the mono-exon subcategory presented a larger difference in length (Supplementary Figure 7.) and exon number (Supplementary Figure 8.) compared to their matched reference transcript than the rest of ISM, ruling out the possibility that these were fragments of initially shorter molecules. These results suggest that ISM transcripts were enriched in 5' end degradation products.

The diversity of TSS and TTS patterns in lrRNA-seq is apparent in the NEXN gene (Supplementary Figure 6.). Although all detected multi-exon transcripts were associated with the same reference transcript model (ENST00000330010.12), they exhibited differences at their 3' and 5' ends that were variously supported by additional data. Two transcripts, classified as 5' fragment ISM, displayed a loss of two exons at their 3' end (PB.1002.4 and PB.1002.6, Supplementary Figure 6.). PB.1002.4 had a shorter last exon that was supported by Quant-seq, a polyA motif, and reference annotation (i.e. shared the TTS in ENST0000440324.5) In contrast, PB.1002.6 had an extended 3' UTR that was not supported by either the reference or Quant-seq, but did contain a known polyA motif. The NEXN gene also had three FSM isoforms that varied in their polyA sites but exhibited higher similarities at the TSS. PB.1002.1, the longest FSM, perfectly matched the reference TTS and was supported by Quant-seq. However, the remaining FSM were 667 bp (PB.1002.2) and 1089 bp (PB.1002.3) shorter than PB.1002.1. Although

polyA motifs were identified for both PB.1002.2 and PB.1002.3, these transcripts lacked Quant-seq evidence, and PB.1002.2 was flagged as a potential intrapriming artifact due to a 20-bp stretch with 90% As found immediately downstream of the TTS, which, together with the location of the polyA motif 39 bp from the 3' end, suggested a TTS annotation of poor quality. Notably, the degree of isoform diversity and TSS/TTS support variability exhibited by the NEXN gene was frequent in the WTC11 lrRNA-seq transcriptome estimated by IsoSeq3.

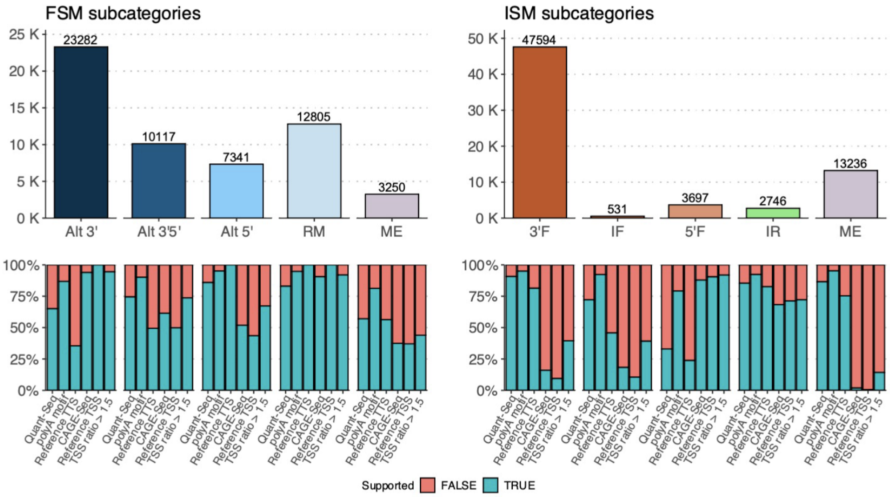

Supplementary Figure 5. SQANTI3 subcategory analysis of WTC11 sample. Supporting evidence for FSM and ISM subcategories at 3' and 5' ends, respectively, by Quant-seq, presence of polyA motifs, and reference annotation for the former, and CAGE-seq, reference annotation, and TSS ratio for the latter.FSM: Full-Splice-Match, ISM: Incomplete-Splice-Match, TSS: Transcription Starting Site, Alt3': Alternative 3' end, Alt3'5': Alternative 3' and 5' end, Alt5': Alternative 5' end, RM: Reference Match, ME: Multi-exon, 3'F: 3' end Fragment, IF: Internal Fragment, 5'F: 5' end Fragment, IR: Intron Retention.

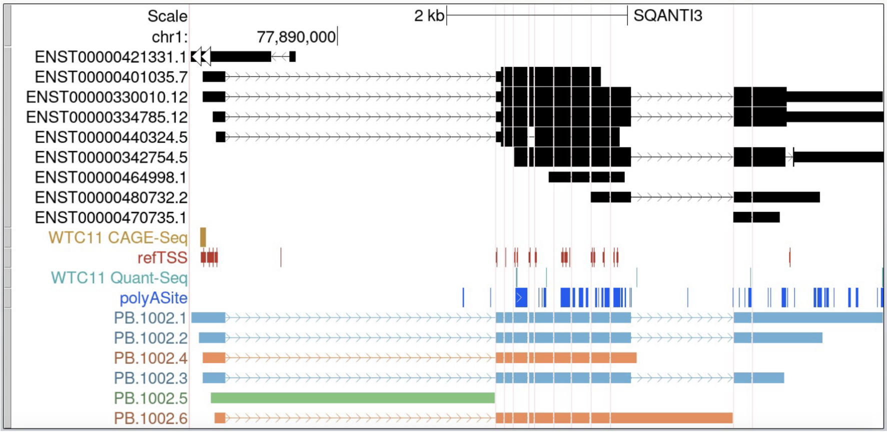

Supplementary Figure 6. SQANTI3 categories and orthogonal support for transcripts of the NEXN gene from the WTC11 PacBio transcriptome. NEXN transcripts are classified as FSM (blue), ISM (orange), and NIC (green) by intron retention. The upper track shows the GENCODE annotation for NEXN (black), while the middle tracks represent CAGE-seq (gold), refTSS (dark red), Quant-seq (light blue), and polyA site (dark blue) annotations. FSM: Full-Splice-Match, ISM: Incomplete-Splice-Match, NIC: Novel-In-Catalog, NNC: Novel-Not-In-Catalog.

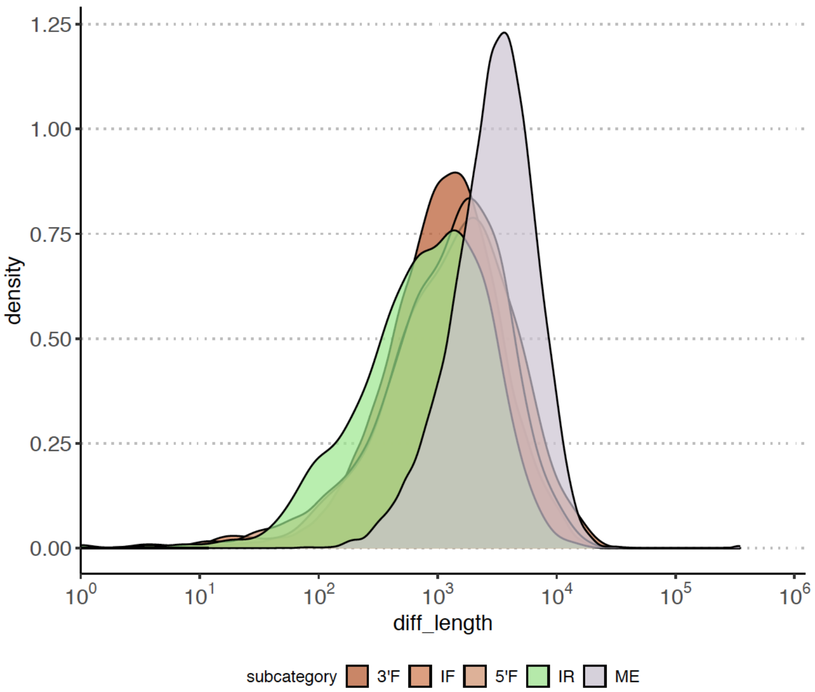

Supplementary Figure 7. Distribution of length differences between ISM isoforms (stratified by subcategory) and their reference counterparts. 3'F: 3'-end Fragment (n=47594), IF: Internal Fragment (n=531), 5'F: 5'-end Fragment (n=3697), IR: Intron Retention (n=2746), ME: Mono-Exon (n=13236).

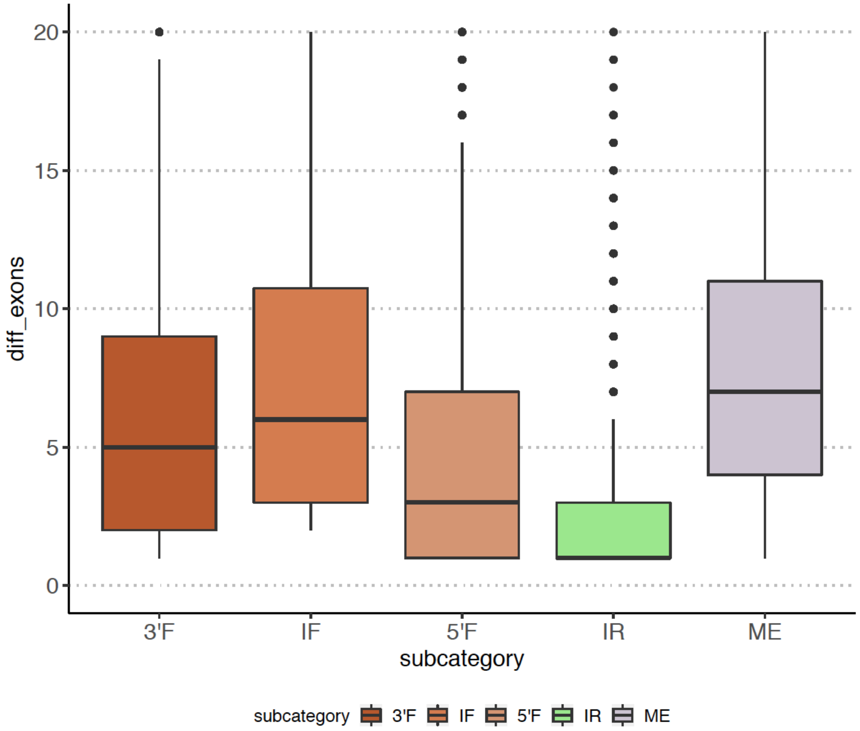

Supplementary Figure 8. Distribution of differences of exon number between ISM isoforms (stratified by subcategory) and their reference counterparts. Boxes indicate median (middle line), 25th (Q1) and 75th (Q3) percentiles (box hinges); whiskers represent min = Q1 − 1.5 · Interquartile Range (IQR) and max = Q3 + 1.5 · IQR; dots constitute outliers. 3'F: 3'-end Fragment (n=47594), IF: Internal Fragment (n=531), 5'F: 5'-end Fragment (n=3697), IR: Intron Retention (n=2746), ME: Mono-Exon (n=13236).

1.4 Supplementary Note 4. Analysis of SQANTI3 Filter performance under a variety of input-data scenarios

SQANTI3 offers a flexible framework for removing artifacts in long-read transcriptomes, based on structural categories and quality descriptors. The Filter module can be adapted to the availability of additional data and the user's preference for using a rules-based approach, where the selection criteria are user-defined, or a machine learning-based strategy, where a classification model is trained.

We used the WTC11 dataset to demonstrate how these filtering alternatives behave in three different scenarios of increasing availability of additional data. The Low Input (LI) scenario represents a situation where only a reference annotation and matching Illumina data are available, which is typical for many studies that use long reads. In the High Input Reference (HIR) setting, additional data are obtained from reference databases such as refTSS and polyA site, resembling the scenario of model species with abundant genomic resources. Finally, the High Input Sample (HIS) setting represents a case where, in addition to the Illumina reads, data such as CAGE and Quant-seq have been obtained (Supplementary Table 2). Besides their differences, HIS, HIR, and LI scenarios shared the same orthogonal evidence coming from short-read sequencing (e.g. SJ coverage) and long-read counts (i.e. number of FL reads used to build each transcript model). To ensure comparable results between rules and ML strategies, we set the rules filter to accept transcript models as long as their SJ, TSS, and TTS were supported by at least one source of external evidence. When running the ML filter, variables used to define the true and false transcript sets were excluded during random forest training to prevent overfitting (see Methods for details). Importantly, given the unavailability of TSS/TTS orthogonal data in the WTC11 LI scenario, built-in TP and TN sets were used in this case, namely FSM in the Reference Match subcategory (TP) and NNC isoforms with at least one non-canonical SJ (TN).

Rules filtering with more input data increased the number of transcript models passing the filter, as expected for the OR configuration (see Methods in main manuscript) used in its design, while the ML approach flagged a higher number of artifacts with increasing amounts of additional data (Supplementary Figure 9.**a**). Specifically, HIS-ML was found to yield the most stringent filtering (173,864 potential artifacts), while HIS-Rules was the most lenient (88,786 potential artifacts). Additionally, we noticed that rules-filtered transcriptomes included a significantly higher number of ISM and NNC transcripts than those obtained using the ML method (Supplementary Figure 9.**a**). This effect was partially mitigated in the LI scenario, where a similar structural category distribution was obtained regardless of the filtering strategy (Supplementary Figure 9.**a**). Interestingly, almost all transcripts flagged as artifacts using SQ3 rules were captured by the ML filter when both were run with the same orthogonal data, with the Low Input scenario being the one with the highest level of agreement (9.**b**).

Supplementary Table 2. Data used in different filtering scenarios.

Sample		Data used during QC, filtering and rescue			
		Matching short-reads	5' end support	3' end support	polyA motifs
WTC11	Low Input (LI)	✓	✗	✗	✗
	High-Input Sample-specific (HIS)	✓	CAGE-Seq	Quant-Seq	✓
	High-Input Reference (HIR)	✓	refTSS database	polyASite database	✓
K562		✓	CAGE-Seq	polyASite database	✓
H1-endoderm differentiation		✓	refTSS database	Quant-Seq	✓

Moreover, a total of 13,969 identified as artifacts in LI settings were supported within HIS and HIR configurations (Supplementary Figure 9.**c**), suggesting that the additional TSS and TTS information is biologically relevant (e.g. PB.1195.1, Supplementary Figure 9.**d**). This underscores the utility of these data sources for validating transcript models. Moreover, when filtering by rules, choosing sample-specific experimental data (HIS) instead of reference databases (HIR) improved the validation of novel TTS, resulting in a decrease in the number of isoforms removed due to the absence of a polyA peak. Specifically, a total of 5,261 isoforms (69.7% of which were ISM) were retained when sample-specific data was used to inform the rules filter (Supplementary Figure 9.**c**), supporting the notion of incompleteness in the current reference annotations.

We next examined the variables selected by the trained ML filter classifier across the different input data contexts. One advantage of this filtering strategy is that it eliminates the need for arbitrary thresholds, automating the decision to accept or reject a transcript model. However, users must still define the true positive (TP) and true negative (TN) transcript sets, which can significantly influence the filtering results. In the LI scenario, where additional evidence for TSS and TTS was lacking, the FSM Reference Match subcategory was used to define TP transcripts, while the TN set was comprised of NNC with non-canonical SJ. These groups differ in SJ quality by definition; however, this configuration did not account for sequencing incompleteness issues, such as false positive TSS/TTS. As a result, attributes related to short-read SJ coverage were the most relevant in the trained random forest model (Supplementary Figure 9.**e**) with minimal contributions from other variables. In contrast, TP and TN sets were defined using CAGE and Quant-seq data for the HIR and HIS scenarios to include transcripts with end variability (see Methods). Metrics such as the TSS ratio, the distance to a known TSS of the same gene, or the number of long reads supporting a transcript model were, therefore, more

relevant for isoform/artifact classification (Supplementary Figure 9.**e**). As a consequence, the ML-HIR and ML-HIS filters removed a greater proportion of ISM transcripts models than ML-LI, while more FSM passed the filter in these cases (Supplementary Figure 9.**a**). This result aligns with the SQANTI3 feature overlap analysis presented in the previous section, confirming the TSS ratio's usefulness as a metric for validation of 5' ends in novel transcripts and demonstrating the existence, at least in human samples, of unannotated combinations of known SJ and TTS/TSS. Finally, differences in the filtering criteria ultimately affect the Rescue process, leading to varying numbers of included genes and isoforms in the curated transcriptomes (Supplementary Figure 9.**f**).

In summary, we show here that the SQANTI3 Filter module is flexible enough to accommodate varying amounts of available data and can be customized to suit the user's curation criteria. Different situations may limit the options available for the user, but also the scope of the research. In the case of building reference transcriptomes, in which transcript models should only be included if enough evidence is found, a ML-filter approach coupled with extensive orthogonal data suits better this purpose. When orthogonal data is scarce or the intention is capturing novel or aberrant isoforms, the Rules strategy allows the user to fine-tune the criteria of exclusion and make the most of the data without annotating clear false transcript models. Finally, we have found that including data supporting 3' and 5' ends in the validation of transcript models can significantly influence the composition of the resulting curated transcriptome. This underscores the limitations of current long-read data and emphasizes the importance of incorporating additional data when defining transcript models using long reads.

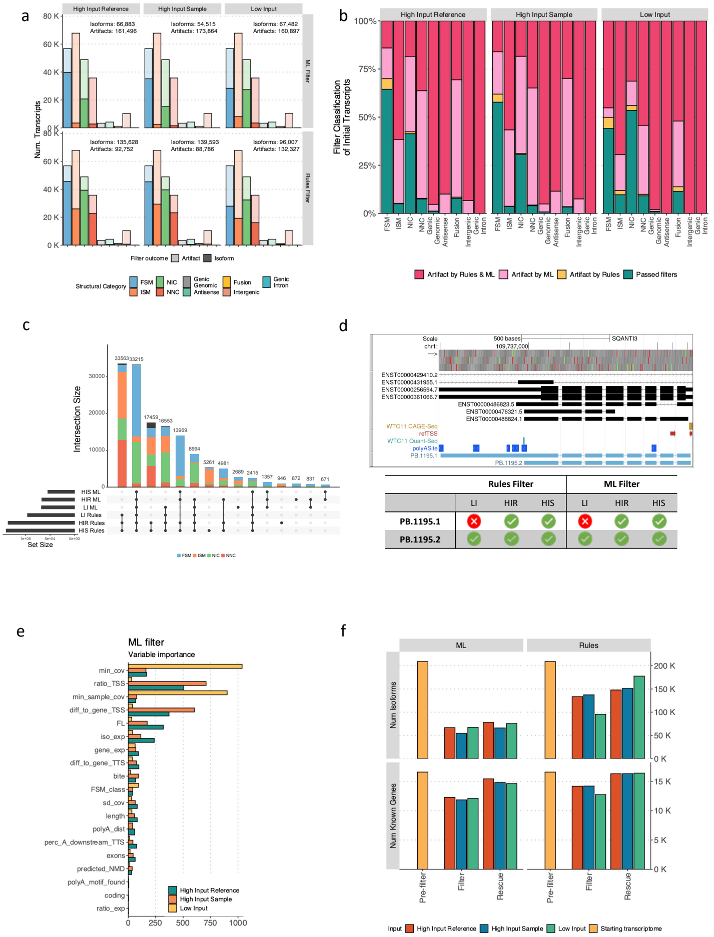

Supplementary Figure 9. SQANTI3 curation. a Artifact and isoform distribution across SQANTI3 structural categories when Rules and ML filters were applied to the WTC11 transcriptome using distinct additional data. **b** Overlap in filtering outcome among filtering methods. **c** Agreement between filtered transcriptomes relied on the strategy and data used. **d** UCSC Genome Browser view of human GST5 gene. Upper track (black) represents the GENCODE annotation for that locus, which includes 5 isoforms for the GST5 gene coded in the negative strand. The bottom track (light blue) shows the LR-defined isoforms identified in the WTC11 sample, while the tracks in between (gold, red, turquoise, and dark blue) are the orthogonal data available to validate those isoforms. The bottom table indicates which filters passed each of the isoforms regarding the informative situation simulated: Low Input (LI), High Input Reference (HIR), and High Input Sample-specific (HIS). **e** Variable importance of ML filter for different input scenarios. **f** Variation in the number of genes and isoforms after Filter and Rescue. FSM: Full-Splice-Match, ISM: Incomplete-Splice-Match, NIC: Novel-In-Catalog, NNC: Novel-Not-In-Catalog, ML: Machine Learning.

1.5 Supplementary Note 5. Application of SQANTI3 pipeline to a dRNA ONT defined transcriptome

The SQANTI3 pipeline is designed to perform data-based QC and curation of transcriptomes, particularly those created using tools with high detection levels and low reference dependence, resulting in a significant proportion of novel isoforms. However, it can be applied to any *de novo* transcriptome, regardless of the sequencing technology or reconstruction pipeline used to generate it. Additionally, SQANTI3 QC can be used to identify and adjust to the algorithmic choices made during transcriptome reconstruction, detecting pipeline-specific sources of bias.

To further demonstrate its wide applicability and versatility, we used SQANTI3 to analyze a long-read transcriptome from a K562 human cell line. The K562 commercial cell line is derived from lymphoblasts from a chronic myelogenous leukemia patient and it is used as a model for immunological research [7]. For the purpose of this study, a lrRNA-Seq K562 dataset obtained using direct RNA (dRNA) Oxford Nanopore Sequencing was retrieved from ENCODE accession ENCSR917JIA. Specifically, we downloaded the reconstructed long-read transcriptome obtained using the TALON pipeline [8], which was available under ENCODE accession ENCFF584GRG. Additional data were retrieved from a variety of experiments involving the same cell line, available at the ENCODE database. Short-read, single-end Illumina data from two K562 samples were obtained from ENCODE accession ENCSR792OIJ. Since single-end data is not supported by SQANTI3 QC short-read processing, Kallisto was run independently and predicted transcript abundances (TPMs) used to build an expression matrix, which was supplied to SQANTI3 QC via `--expression` argument. CAGE-Seq data was downloaded from ENCODE accession ENCSR000CJN and included 9,248 peaks. Given the unavailability of K562 Quant-Seq datasets, the reference database described in the previous section (polyASite v2.0, human) was used for TTS validation, together with the same list of human polyA motifs. To obtain the read counts associated with each transcript model, we parsed the information from the transcript quantification file under the ENCODE accession ENCFF668BLB. All these data were used as input for SQANTI3 QC.

The TALON transcriptome contained 336,445 isoforms, 95% of them belonging to 51,843 known genes. SQANTI3 identified a relatively large proportion of FSM (∼60%, Supplementary Figure 10.**a**). Of these, the vast majority (85.5%) were classified within the Reference Match subcategory (Supplementary Figure 10.**b**, upper panel), which involved 163,319 reference transcripts, roughly two-thirds of the entire GENCODE annotation. Further analysis revealed that one-third of all identified FSM (33.1%) had Illumina support across all SJ and 11.4% showed expression above 1 TPM (Supplementary Figure 10.**b**, lower panel). These results suggest that the TALON transcriptome reconstruction process was largely guided by the reference annotation, leading to moderate support by orthogonal data.

We next applied the two strategies within the SQANTI3 Filter module to the K562 dRNA ONT transcriptome. Because of the large number of FSM with poor orthogonal support, we adjusted the rules filter to require SJ coverage of at least 3 short-reads in all structural categories, including FSM. Isoforms were additionally removed if mono-exonic or flagged as intrapriming or RT-Switching. For the ML filter, the TP and TN sets were defined as detailed in Methods for the WTC11 dataset.

As a result of rules filtering, the vast majority of FSM and NNC isoforms were flagged as potential artifacts, mainly due to their low expression values and the presence of at least one SJ lacking short-read coverage (Supplementary Figure 10.**c**). Moreover, the number of peaks in the available CAGE-seq dataset was low limiting the utility of this information for TSS assessment (Supplementary Figure 10.**c**). However, this did not prevent the validation of novel isoforms reported by TALON and after filtering by rules, the NIC and NNC categories accounted for about ∼15% of all filter-passing transcript models, exhibiting relatively high-quality attributes (Supplementary Figure 10.**c**). The ML-filtered transcriptome, on the other hand, contained a remarkably high proportion of FSM and a comparatively lower number of ISM, NIC, and NNC (Supplementary Figure 10.**d**). We attributed this result to the reference-biased and poor SJ coverage of the TALON FSM transcripts, together with the limitations of the CAGE-seq data, both of which compromised the definition of the TP set. In fact, most filter-passing FSM had at least one SJ lacking coverage and low short-read-based expression values (Supplementary Figure 10.**d**), suggesting poor support of their presence in the K562 samples. Therefore, we concluded that a tailored rules filter would be a more suitable approach in this case, as it yielded a more data-supported transcriptome. Similarly, the rescue strategy can be adapted to the characteristics of this dataset. The *automatic rescue* was in this case not advisable, as the high number of FSM flagged as artifacts were recovered by reference transcripts with equally poor support (Supplementary Figure 10.**e**). Instead, the detailed information included in the output of the rescue module can be used to define an *ad hoc* rescue criteria. In this case, we opted to only recover rescue-by-mapping isoforms, as these are supported by complementary data. The information retrieved by the SQ3 rescue module, therefore, allows for a critical evaluation of the properties of each specific transcriptome, enabling users to make decisions tailored to their dataset.

Overall, this example demonstrates the versatility of the SQANTI3 toolkit for designing a transcriptome curation strategy. Users have the ability to customize each step of the pipeline to meet their specific data needs. In the case of the TALON K562 dRNA ONT transcriptome, FSM were in high proportion but often lacked additional evidence of expression, compromising the definition of a high-quality true positive transcript model set. Therefore, rather than using the ML approach, the rules filter was adjusted based on the information obtained through SQANTI3 QC. Additionally, the information in the output

of the rescue step was used to recover only well-supported transcript models. These examples also underscore the value of complementary data in the curation process and the need for a critical assessment of each dataset.

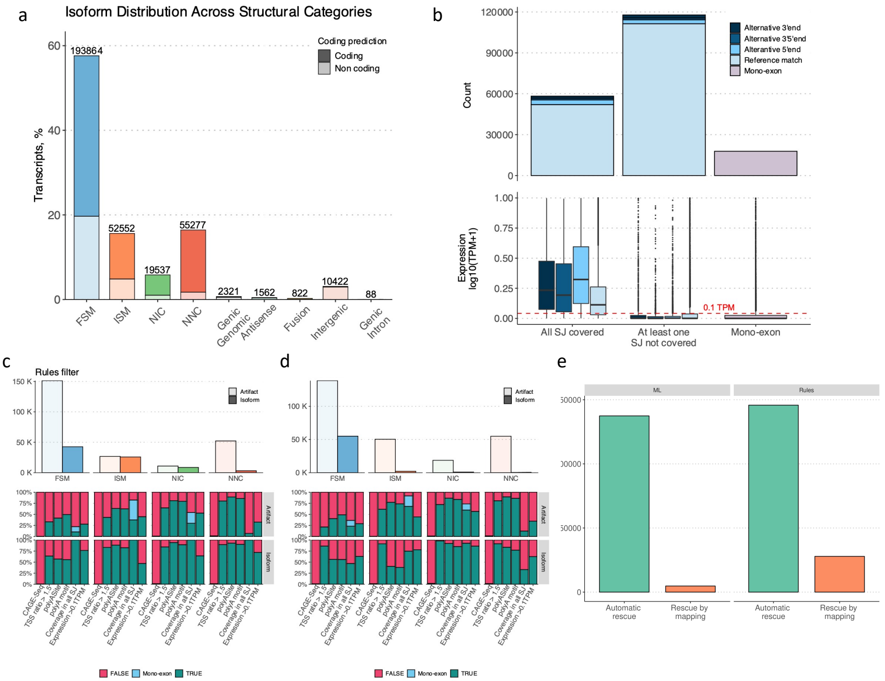

Supplementary Figure 10. QC and filtering of the K526 dataset. a, Distribution of structural categories in the K562 TALON-defined transcriptome. **b**, Characterization FSM transcript models of the K562 TALON-defined transcriptome. Incidence of artifacts detected across structural categories according to SQANTI3 filter using a **c** rules or a **d** ML approach. **e** Number of transcripts incorporated into the K562 transcriptome after SQANTI3 rescue, shown by filter strategy and separated by whether they were obtained during *automatic rescue* or after rescue-by-mapping. Boxes indicate median (middle line), 25th (Q1) and 75th (Q3) percentiles (box hinges); whiskers represent min = Q1 − 1.5 · Interquartile Range (IQR) and max = Q3 + 1.5 · IQR; dots constitute outliers. FSM: Full-Splice-Match, ISM: Incomplete-Splice-Match, NIC: Novel-In-Catalog, NNC: Novel-Not-In-Catalog, SJ: Splice Junction.

References

[1] de la Fuente, L., Arzalluz-Luque, A., Tardáguila, M., del Risco, H., Martí, C., Tarazona, S., Salguero, P., Scott, R., Lerma, A., Alastrue-Agudo, A., Bonilla, P., Newman, J.R.B., Kosugi, S., McIntyre, L.M., Moreno-Manzano, V., Conesa, A.: tappAS: a comprehensive computational framework for the analysis of the functional impact of differential splicing. Genome Biology 21(1), 119 (2020).

[2] Siller, R., Naumovska, E., Mathapati, S., Lycke, M., Greenhough, S., Sullivan, G.J.: Development of a rapid screen for the endodermal differentiation potential of human pluripotent stem cell lines. Scientific Reports 6(1), 37178 (2016).

[3] Grillo, G., Turi, A., Licciulli, F., Mignone, F., Liuni, S., Banfi, S., Gennarino, V.A., Horner, D.S., Pavesi, G., Picardi, E., Pesole, G.: UTRdb and UTRsite (RELEASE 2010): a collection of sequences and regulatory motifs of the untranslated regions of eukaryotic mRNAs. Nucleic Acids Research 38, 75–80 (2010).

[4] Baumgart, E., Vanhooren, J.C., Fransen, M., Marynen, P., Puype, M., Vandekerckhove, J., Leunissen, J.A., Fahimi, H.D., Mannaerts, G.P., van Veldhoven, P.P.: Molecular characterization of the human peroxisomal branched-chain acyl-CoA oxidase: cDNA cloning, chromosomal assignment, tissue distribution, and evidence for the absence of the protein in zellweger syndrome. Proceedings of the National Academy of Sciences of the United States of America 93(24), 13748–1375 (1996).

[5] Russell, L., Garrett-Sinha, L.A.: Transcription factor ets-1 in cytokine and chemokine gene regulation. Cytokine 51(3), 217–22 (2010).

[6] Caron, C., Pivot-Pajot, C., van Grunsven, L.A., Col, E., Lestrat, C., Rousseaux, S., Khochbin, S.: Cdyl: a new transcriptional co-repressor. EMBO reports 4(9), 877–882 (2003).

[7] Klein, E., Ben-Bassat, H., Neumann, H., Ralph, P., Zeuthen, J., Polliack, A., Vánky, F.: Properties of the k562 cell line, derived from a patient with chronic myeloid leukemia. International Journal of Cancer 18(4), 421–431 (1976).

[8] Wyman, D., Balderrama-Gutierrez, G., Reese, F., Jiang, S., Rahmanian, S., Forner, S., Matheos, D., Zeng, W., Williams, B., Trout, D., England, W., Chu, S.-H., Spitale, R.C., Tenner, A.J., Wold, B.J., Mortazavi, A.: A technology-agnostic long-read analysis pipeline for transcriptome discovery and quantification. Peprint at: https://www.biorxiv.org/content/10.1101/672931v2.

2 Supplementary Methods

2.1 SQANTI3 QC

2.1.1 STAR

If matching short-reads are provided to SQANTI3, it will run internally STAR and Kallisto to generate orthogonal information, which eventually serves to characterize the transcriptome and validate (or reject) transcript models during the curation process.

The exact commands used are the following:

```
STAR --runThreadN <num cpus> \
    --runMode genomeGenerate \
    --genomeDir <index_dir> \
    --genomeFastaFiles <fasta_genome> \
    --outTmpDir <index_dir_tmp>

STAR --runThreadN <num cpus> --genomeDir <index_dir> \
    --readFilesIn <read1> <read2> \
    --outFileNamePrefix <sample_prefix> \
    --alignSJoverhangMin 8 \
    --alignSJDBoverhangMin 1 --outFilterType BySJout \
    --outSAMunmapped Within \
    --outFilterMultimapNmax 20 \
    --outFilterMismatchNoverLmax 0.04 \
    --outFilterMismatchNmax 999 --alignIntronMin 20 \
    --alignIntronMax 1000000 --alignMatesGapMax 1000000 \
    --sjdbScore 1 --genomeLoad NoSharedMemory \
    --outSAMtype BAM SortedByCoordinate --twopassMode Basic
```

2.1.2 Kallisto

We also implemented Kallisto's quantification inside SQANTI3 QC pipeline, so if paired-end data is input, Kallsito will create an index with the sequences of the transcript models being evaluated and quantify them using short reads. The exact commands are these:

```
kallisto index -i <kallisto_index> \
    <corrected_fasta> --make-unique

kallisto quant -i <kallisto_index> \
    -b 100 -t <num cpus> <read1> <read2>
```

The process is fully automated for pair-end data, but in some cases, like the K562 dataset where only single-end data is available, Kallisto (or any other quantification tool) can be run previously and provide its output to SQANTI3

via `--expression` parameter. In the K562 case, as the length of the fragments cannot be estimated with single-end reads, the same command described previously was run with some extra parameters based on the metadata available in the dataset ENCODE site:

```
kallisto quant -i <kallisto_index> \
    -b 100 -t <num cpus> --single \
    --rf-stranded -l 240 -s 30 <read1>
```

2.2 Running IsoSeq3 from subreads to transcriptome

2.2.1 CCS

```
subreads="subreads/"$sample".bam"
ccs=$sample".ccs.bam"

ccs $subreads $ccs --min-rq 0.9
```

2.2.2 lima and refine

```
names="cDNA_subreads_names.txt"
sample="WTC11.cDNA"
primers="primer.fasta"
fofn=$sample".flnc.fofn"

while read -r line
    do
    ccs="subreads/"$line".ccs.bam"
    fl=$line".fl.bam"
    lima $ccs $primers $fl --isoseq --peek-guess -j8

    fl_primers=$line".fl.Clontech_5p--Clontech_3p.bam"
    flnc="IsoSeq3_output/"$line".flnc.bam"
    isoseq3 refine $fl_primers $primers $flnc --require-polya -j 8
```

2.2.3 IsoSeq3 cluster and collapse

```
clustered=$sample".clustered.bam"
isoseq3 cluster $fofn $clustered --verbose --use-qvs -j 8

refGenome="lrgasp_grch38_sirvs.fasta"
hq_isoforms=$sample".clustered.hq.fasta"
hq_sam=$sample".clustered.hq.sam"
hq_sorted_sam=$sample".clustered.hq.sorted.sam"
minimap2 -ax splice -t 8 -uf --secondary=no -C5 $refGenome

$hq_isoforms > $hq_sam
```

```
sort -k 3,3 -k 4,4n $hq_sam > $hq_sorted_sam
collapse_isoforms_by_sam.py --input $hq_isoforms -s $hq_sorted_sam \
    --dun-merge-5-shorter -o $sample

get_abundance_post_collapse.py $sample".collapsed"
$sample".clustered.cluster_report.csv"
```

2.3 Running SQANTI3 QC for the WTC11 transcriptome

2.3.1 Reference genome and annotation

```
refGenome="lrgasp_grch38_sirvs.fasta"
refGTF="lrgasp_gencode_v39_annotation_sirvs.human.gtf"
```

2.3.2 Short-read support

In order to speed up the data analysis and make it more consistent, we only ran once the mapping step with STAR using the command lines described above. Then, we took the resulting SJ.out.tab and BAM files to the rest of the analysis and input into SQANTI3 using the `-c` and `{SR_bam` arguments.

2.3.3 PolyA motif list

The following list of polyA motifs was used as input to SQANTI3 via the `--polyA_motif_list` argument:

```
aataaa
attaaa
agtaaa
tataaa
cataaa
gataaa
aatata
aataca
aataga
aaaaag
actaaa
aagaaa
aatgaa
tttaaa
aaaaca
ggggct
```

2.4 Running SQANTI3 QC for the Low Input (LI) scenario

```
python sqanti3_qc.py $sample $refGTF $refGenome \
```

```
    --fl_count $fl \
    -c $cov --short_reads $SR \
    --SR_bam $SR_bam \
    -o WTC11_cDNA.LI -d $out_dir -t8 \
    --report both -t 8
```

2.5 Running SQANTI3 QC for High Input Sample (HIS) and Reference (HIR) scenarios

```
refTSS_peaks=human.refTSS_v3.1.hg38.bed # or WTC11_CAGE.filtered.bed
polyAsite=polyASite_atlas.2.0.GRCh38.96.bed

python sqanti3_qc.py $sample $refGTF $refGenome \
    --CAGE_peak $refTSS_peaks --fl_count $fl \
    --polyA_motif_list $polyA_motifs \
    -c $cov --short_reads $SR \
    --polyA_peak $polyA_sites --SR_bam $SR_bam \
    -o WTC11_cDNA.HIR -d $out_dir -t8 \
    --report both -t 8
```

2.6 Running SQANTI3 QC for the K562 transcriptome

```
cage_peaks=CAGE_data.ENCFF981XPE.bed
polyA_sites=polyASite_atlas.2.0.GRCh38.96.bed

python sqanti3_qc.py $sample $refGTF $refGenome \
    --CAGE_peak $cage_peaks --fl_count $fl \
    --polyA_motif_list $polyA_motifs \
    --short_reads $SR --expression $kallisto_expression \
    --polyA_peak $polyA_sites_ref \
    -o K562_replicate1.SQ3 -d $out_dir -t8 \
    --report both
```

2.7 Running SQANTI3 QC for the H1-endoderm transcriptome

```
isoAnnot_gff4="Homo_sapiens_Gencode_v39.zip"

python sqanti3_qc.py $sample $refGTF $refGenome \
    --CAGE_peak $refTSS_peaks --fl_count $fl \
    --polyA_motif_list $polyA_motifs \
    --short_reads $SR \
    --polyA_peak $polyA_sites -e $expression_files \
    -o real_case.SQ3 -d $out_dir -t8 \
    --report both -t 8 --isoAnnotLite --gff3 $isoAnnot_gff3
```

2.8 Running SQANTI3 QC for reference transcriptomes

This is the command used to characterize the reference annotation regarding to the available data in each scenario. The `cage_peaks_ref` and `polyA_sites` variables will change depending on the dataset.

```
python sqanti3_qc.py $refGTF $refGTF $refGenome \
    --CAGE_peak $cage_peaks_ref \
    --polyA_motif_list $polyA_motifs \
    --short_reads $SR --genename \
    --polyA_peak $polyA_sites \
    -o reference.SQ3 -d $out_dir -t8 \
    --report skip -t 8 --force_id_ignore --min_ref_len 0
```
