# Supplementary material for: SQANTI3: curation of long-read transcriptomes for accurate identification of known and novel isoforms
Source: Nat Methods. 2024 Mar 20;21(5):793–7. doi: 10.1038/s41592-024-02229-2 (PMC11093726; doi:10.1038/s41592-024-02229-2)
Supplement: Supplementary file 2 — Reporting Summary [file 41592_2024_2229_MOESM2_ESM.pdf]

nature portfolio

Corresponding author(s): Ana Conesa Cegarra (ana.conesa@csic.es)

Last updated by author(s): 20/12/2023

Reporting Summary

Nature Portfolio wishes to improve the reproducibility of the work that we publish. This form provides structure for consistency and transparency in reporting. For further information on Nature Portfolio policies, see our Editorial Policies and the Editorial Policy Checklist.

Statistics

For all statistical analyses, confirm that the following items are present in the figure legend, table legend, main text, or Methods section.

n/a	Confirmed	
☐	☒	The exact sample size (*n*) for each experimental group/condition, given as a discrete number and unit of measurement
☐	☒	A statement on whether measurements were taken from distinct samples or whether the same sample was measured repeatedly
☐	☒	The statistical test(s) used AND whether they are one- or two-sided<br>*Only common tests should be described solely by name; describe more complex techniques in the Methods section.*
☐	☒	A description of all covariates tested
☐	☒	A description of any assumptions or corrections, such as tests of normality and adjustment for multiple comparisons
☐	☒	A full description of the statistical parameters including central tendency (e.g. means) or other basic estimates (e.g. regression coefficient) AND variation (e.g. standard deviation) or associated estimates of uncertainty (e.g. confidence intervals)
☐	☒	For null hypothesis testing, the test statistic (e.g. *F*, *t*, *r*) with confidence intervals, effect sizes, degrees of freedom and *P* value noted<br>*Give P values as exact values whenever suitable.*
☒	☐	For Bayesian analysis, information on the choice of priors and Markov chain Monte Carlo settings
☐	☒	For hierarchical and complex designs, identification of the appropriate level for tests and full reporting of outcomes
☒	☐	Estimates of effect sizes (e.g. Cohen's *d*, Pearson's *r*), indicating how they were calculated

Our web collection on statistics for biologists contains articles on many of the points above.

Software and code

Policy information about availability of computer code

Data collection	No software was used for data collection
Data analysis	https://github.com/ConesaLab/SQANTI3

For manuscripts utilizing custom algorithms or software that are central to the research but not yet described in published literature, software must be made available to editors and reviewers. We strongly encourage code deposition in a community repository (e.g. GitHub). See the Nature Portfolio guidelines for submitting code & software for further information.

Data

Policy information about availability of data

All manuscripts must include a data availability statement. This statement should provide the following information, where applicable:

- Accession codes, unique identifiers, or web links for publicly available datasets
- A description of any restrictions on data availability
- For clinical datasets or third party data, please ensure that the statement adheres to our policy

Data used in this manuscript is publicly available under the following accession codes: for WTC11 analysis lrRNA-Seq (ENCODE ENCSR507JOF), srRNA-Seq (ENCODE ENCSR673UKZ), CAGE-Seq (GEO GSE185917) and Quant-Seq (ENCODE ENCSR322MWL); for K562 analysis it was used ENCODE ENCSR917JIA experiment data with its long-read-based transcriptome (ENCODE ENCFF584GRG), srRNA-Seq (ENCODE ENCSR792OIJ) and CAGE-Seq data (ENCODE ENCSR000CJN); and for H1-endoderm analysis data included lrRNA-Seq ENCODE ENCSR271KEJ (H1-hESC) and ENCSR127HKN (H1-DE), srRNA-Seq ENCODE ENCSR588EJX (H1-hESC) and ENCSR266XAJ (H1-

DE), and Quant-Seq ENCODE ENCSR198UNH (H1-hESC) and ENCSR198UNH (H1-DE). Reference data from databases such as polyASite and refTSS was used to validate 3' and 5'-ends. IsoAnnot annotation for human used InterProScan (https://www.ebi.ac.uk/interpro/), UniProt (https://www.uniprot.org/) and Modi-DB (https://mobidb.org/) databases to functionally annotate reference transcripts.

All the files used to generate the results in this paper are publicly accessible at http://conesalab.org/SQANTI3/. For an easier exploration of WTC11 transcript models identified with IsoSeq3 and their characterization with SQANTI3, a specific and public Track hub was generated for the UCSC Genome Browser (hub URL: http://conesalab.org/SQANTI3/WTC11/SQANTI3_hub/hub.txt), including the orthogonal data used for validation. We make a special emphasis on the availability of the results of hESC H1 cells to endoderm differentiation in a ready-to-use format on tappAS http://conesalab.org/SQANTI3/H1_endo/tappAS_files/).

Human research participants

Policy information about studies involving human research participants and Sex and Gender in Research.

Reporting on sex and gender	NA
Population characteristics	NA
Recruitment	NA
Ethics oversight	NA

Note that full information on the approval of the study protocol must also be provided in the manuscript.

Field-specific reporting

Please select the one below that is the best fit for your research. If you are not sure, read the appropriate sections before making your selection.

☒ Life sciences ☐ Behavioural & social sciences ☐ Ecological, evolutionary & environmental sciences

For a reference copy of the document with all sections, see nature.com/documents/nr-reporting-summary-flat.pdf

Life sciences study design

All studies must disclose on these points even when the disclosure is negative.

Sample size	Three different samples (WTC11, K562 and H1-endoderm cells) were obtained from public data bases with their corresponding replicates. No statistical analysis involving the utilization of different cell lines was performed in the study and these 3 samples were used to illustrate wide applicability. Statistical analyses evaluating association between transcript quality descriptors such as CAGE-peak coverage, polyA motif, etc, were performed on the whole transcriptome datasets with over 100,000 transcripts, that act as the number of observations or sample size for these test and is considered sufficient for the purposes of these tests.
Data exclusions	Data wasn't excluded in any case
Replication	Experimentation per se was not used in this study and the utilization of multiple public datasets was sufficient for the nature of the analyses performed. As indicated in the Sample Size section, the utilization of the whole transcriptome provided sufficient number of observations to support the statistical analyses.
Randomization	No randomization was done except for the selection of TP and TN sets. Transcript models were randomly selected following the criteria described in the paper for each data availability scenario simulated in order to run the ML-filtering algorithm.
Blinding	In general blinding was not applicable to the study. Predictions were performed without knowledge of the ground truth to be able to compute performance metrics for the SQANTI3 curation strategy.

Reporting for specific materials, systems and methods

We require information from authors about some types of materials, experimental systems and methods used in many studies. Here, indicate whether each material, system or method listed is relevant to your study. If you are not sure if a list item applies to your research, read the appropriate section before selecting a response.

Materials & experimental systems

n/a	Involved in the study
☒	☐ Antibodies
☒	☐ Eukaryotic cell lines
☒	☐ Palaeontology and archaeology
☒	☐ Animals and other organisms
☒	☐ Clinical data
☒	☐ Dual use research of concern

Methods

n/a	Involved in the study
☒	☐ ChIP-seq
☒	☐ Flow cytometry
☒	☐ MRI-based neuroimaging
